# Family drawing for assessing attachment in children: Weaknesses and strengths

**DOI:** 10.3389/fpsyg.2022.980129

**Published:** 2022-09-12

**Authors:** Cecilia Serena Pace, Stefania Muzi, Francesca Vizzino

**Affiliations:** University of Genoa, Genoa, Italy

**Keywords:** family drawing, attachment representations, children, ABCD system, Fury’s global rating scales, psychometric properties, culture-fair method

## Introduction

The drawing represents a projective technique widely used by clinical and developmental psychologists to access a child’s inner world (Falk, 1981; Pianta and Longmaid, 2010; Procaccia et al., 2014). Particularly, the Family Drawing (FD) developed by the psychoanalyst Corman (1967) is one of the most used tests to explore perceptions of relationships in children aged 5−⁣−15 years and, thanks to Kaplan and Main (1986) and Fury (1996) contributions, it can also assess children’s attachment representations.

The Family Drawing (FD) with an attachment-based coding system includes three levels: a checklist of individual markers (Kaplan and Main, 1986), which comprises 24 features of drawing whose presence or absence is assessed; four global attachment classifications, *i.e.*, ABCD classifications, namely, Secure (B), Avoidant (A), Ambivalent (C), and Disorganized (D), assigned based on a global evaluation of marker combinations (Kaplan and Main, 1986); eight global rating scales scored from 1 (absent) to 7 (very high) points, added by Fury (1996), who also redesigned criteria of assignment of ABCD categories considering both markers and scale scores. Table 1 details the characteristics of these attachment-based coding systems.

**TABLE 1:** Three levels of attachment-based coding system on Family Drawing.

24 Individual markers^a^ (Kaplan and Main, 1986)	Four classifications (Kaplan and Main, 1986)	Eight global rating scales^b^ (Fury, 1996)
**Avoidant markers (A)** (1) Lack of color (2) Child positioned far apart from mother (3) Omission of mother or child (4) Lack of individuation of family members (5) Arms downward, close to the body (6) Exaggeration of heads (7) Disguised family members	**Secure (B)** Drawings show centered, grounded and completed figures with open arms; high *family pride/happiness* and low *global pathology*.	**Security (B) scales:** (1) *Vitality/Creativity:* the child’s emotional investment in drawing is reflected in creativity, detail, and embellishment; (2) *Family Pride/Happiness*: a child’ sense of belonging to the family troupe;
**Ambivalent markers (C)** (8) Figures separated by barriers (9) Figures crowded or overlapping (10) Unusually small figures (11) Unusually large figures (12) Exaggeration of body part (13) Exaggeration of hands/arms (14) Exaggeration of facial features (15) Figures on the corner of the page	**Insecure-avoidant (A)** Drawings are characterized by distance between family members, uncolored or uncompleted figures (e.g., without arms), and an emphasis on invulnerability is expressed by happy face; high *emotional distance/isolation* and *tension/anger.*	**Avoidant (A) scales:** (3) *Emotional Distance/Isolation*: a sense of loneliness perceived by the child reflected in masked expressions of anger, neutral or negative affects, distance between mother and child (4) *Tension/Anger:* figures without color and detail or scribbled or crossed out;
**Insecurity markers (A,C)** (16) Lack of background detail (17) Figures not grounded (“floating”) (18) Incomplete figures (19) Mother not feminized (20) Males and females undifferentiated by gender (21) Neutral/negative facial affect	**Insecure-ambivalent** Drawings show vulnerability in family relations, with crowded or overlapping figures and a large or small figures; high *Vulnerability* and *Role Reversal*	**Ambivalent (C) scales:** (5) *Vulnerability:* placement of figures on the page and exaggeration of body parts; (6) *Role Reversal:* size or roles of drawing figures.
**Disorganized markers (D)** (22) False starts (23) Scrunched figures (24) Unusual signs, symbols, or scenes	**Disorganized** Drawing is characterized by confusing and fluctuating figures with unusual signs and symbols; high *bizarreness/dissociation* and *global pathology*	**Disorganized (D) scales:** (7) *Bizarreness/Dissociation*: unusual symbols, signs, and fantasy themes; 8) *Global Pathology*: which is reflected in the global organization of drawing, including, for example, the completeness of figures, the use of color, presence of details, affect, and background scene.

^a^Coded in eight dimensions: the degree of movement present in the figures, the identification of the figures, the completeness of the human forms represented, the quality of the smile, the size of the figures, the centrality of the figures in the sheet, and the global impression of vulnerability/invulnerability.

^b^Main categories are assigned based on high scores in pattern scales and global impressions of balance and enhancement of affective ties (Secure); emotional indifference and coldness (Avoidant), isolation from the family group or fear and worry (Ambivalent), chaos, confusion, and anxiety (Disorganized).

The administration of the FD follows the procedure described by Kaplan and Main (1986) and Fury et al. (1997). It requires an 8.5 × 11 cm white paper placed horizontally in front of the child and a set of colored markers. Different from other methods, such as the gold-standard Strange Situation Procedure (SSP; Ainsworth et al., 1978; van Ijzendoorn et al., 1992), the child is not subjected to stressful situations or stimuli but is asked to draw a picture of his/her family. As a projective open-ended technique, no other information is given. When the drawing has been completed, the child is asked to identify all the people in the drawing and explain their relationship with the child. The assessment is made according to some indicators that suggest certain patterns of attachment (*e.g.*, lack of color or distance between family members suggests an avoidant attachment, and unusually small figures or exaggeration of body parts suggests an ambivalent attachment, as detailed in Table 1).

Because drawing is non-verbal communication, FD is thought to be useful when it is not possible or unreliable to rely on verbal communication. For example, with internationally low-language proficiency adopted children (Pace et al., 2015), children with selective mutism, or other clinical conditions that involve language difficulty (*e.g.*, too fast and confused speech in ADHD, Clarke et al., 2002), as well as when the child may be frightened, reluctant or not used to communicate about family relationships, as in the case of abused children, children exposed to family problems (Leon and Rudy, 2005), or children of depressed mothers (Fihrer and McMahon, 2009).

As shown by a recent systematic review and meta-analysis on this topic (Pace et al., 2021), the FD has been increasingly used to assess attachment in the last decade, either with community, clinical, or at-risk children from 5 to 13 years old. Most of the studies employed a double coding system, *i.e.*, both the Main and Kaplan’s ABCD classifications, and Fury’s scales (detailed in Table 1), with the former being suggested as maybe less accurate than the latter, especially in clinical and at-risk samples. Other findings also suggested a culture-fair potentiality of FD in non-Western collectivistic cultures, because of the possibility offered by this method to assess attachment representations toward multiple attachment figures in the drawing, overcoming the exclusive focus on mother and father typical of Western cultures.

In sum, the scarce studies and review findings opened questions concerning FD psychometric properties and culture-fair potential in non-Western collectivistic cultures, which still need more investigation.

### Sub-section 1: How are the psychometric properties of the Family Drawing attachment-based coding systems?

Focusing on the psychometric properties of the FD with an attachment-based coding system, studies provided values of *inter-rater reliability* being from acceptable to good for ABCD classifications, *i.e.*, Cohen’s *k* between 0.64 and 0.80 (Madigan et al., 2003; Pianta and Longmaid, 2010; Behrens and Kaplan, 2011), and good to optimal for Fury’s global scales, *i.e.*, Cohen’s *k* between 0.75 and 1.00 (Fury et al., 1997; Madigan et al., 2003; Pianta and Longmaid, 2010), and Pearson’s *r* from 0.54 to 0.95 (Fury et al., 1997; Madigan et al., 2003). These results suggest if independent and blinded evaluators of the FD employ the same parameters, they usually assign the same classification or score, which indicates that the coding guidelines of both ABCD and Fury scale systems are clear and well-explained, understandable by different possible raters.

Regarding FD *discriminant validity*, some studies found relations between children’s IQ and both ABCD categories (Pianta and Longmaid, 2010) and Fury scales scores in community and at-risk children (Dallaire et al., 2012). However, other studies did not find any relationship between FD and IQ or children’s fine motor skills (Fury, 1996; Madigan et al., 2003; Leon et al., 2007; Pace et al., 2015). Surprisingly, no studies explored the discriminant validity of the child’s ability to draw.

Concerning the *concurrent validity*, studies in different populations of children (*i.e.*, community, clinical, adopted, *etc.*) showed the attachment-based FD results partially converged with those of the gold-standard SSP and the Manchester Child Attachment Story Task (MCAST; Goldwyn et al., 2000), a completion task used to assess attachment representations in 4–8 years old children and rated both through four ABCD classifications and 1-to 9-point scales (Jin et al., 2018; Pace et al., 2020; Kallitsoglou et al., 2021).

Particularly, the results of the meta-analyses with the SSP (van Ijzendoorn et al., 1992) and with FD (Pace et al., 2021) converge toward the higher prevalence of secure classifications over the insecure ones in community children and security scores as the highest in Fury’s scales. Moreover, with both instruments, the community children showed higher security than clinical and at-risk ones. However, the meta-analytic rate of C categories with the Family Drawing ABCD system was markedly higher than the rate in the meta-analysis of SSP, so the authors have suggested a possible overestimation of the C pattern employing the Kaplan and Main (1986) system on FD which should be further investigated. The authors have also observed higher convergence of results between SSP and Fury scales than with the ABCD system (Pace et al., 2021).

Few studies explored the convergence of FD results with those of the MCAST (Goldwyn et al., 2000), reporting contrasting results across samples, which suggest further investigation. Specifically, Jin et al. (2018) suggested convergence in both classifications and scales of community and (especially) clinical children. Pace et al. (2020) reported convergence of more scales in communities than in at-risk children, and Kallitsoglou et al. (2021) suggested no convergence of scales among the communities. Overall, these results are too heterogeneous to assume that FD can be as trustable as other more validated attachment measures in assessing attachment, and further studies are needed.

Regarding *clinical validity*, several studies showed that FD attachment-based coding systems can discriminate against higher attachment insecurity in clinical and at-risk children (*i.e.*, ADHD, adopted, abused, etc.) using Fury’s global scales (Clarke et al., 2002; Dallaire et al., 2012; Pace et al., 2015; Howard et al., 2017; Jin et al., 2018). If future psychometric studies will prove the reliability of the results obtained with the FD, these findings suggest practitioners employ the FD as a simple, economic, and useful method to assess attachment in vulnerable groups.

Last but not least, Pace et al. (2021) rated the quality of studies included in the systematic review, revealing fair to moderate quality for most of them, which mostly did not check the influence of demographics on results which should be further investigated.

### Sub-section 2: Can the Family Drawing be considered a culture-fair method to assess attachment in children around the world?

As detailed in the review by Pace et al. (2021), attachment of pre- and school-aged children has been mainly evaluated through observational procedures, *e.g.*, the SSP, or narrative completion tasks, *e.g.*, the MCAST. Both of them have limitations at this age: the former because it is mostly based on behaviors that children show with their parents up to 2 years of age and the latter because results can be influenced by the child’s verbal abilities or cultural stereotypes transmitted through language (Burgers and Beukeboom, 2020).

Drawing instead has been reported as a culture-fair option to assess psychological abilities, *e.g.*, (Weiss, 1980), and the cited meta-analysis seems to recognize this potential also in FD, as cultural differences did not completely overlap with those hypothesized based on general literature (Mesman et al., 2016). Specifically, the distribution of any ABCD category did not significantly differ between community children from Western (*i.e.*, Canada, United States, Italy, and Greece) or non-Western (*i.e.*, Israel, Japan, Korea, and Cameroon) cultural backgrounds. However, differences emerged employing Fury’s scales, with Western community children scoring higher than non-Western ones on all scales related to insecure patterns, revealing a counterintuitive trend. A limitation of this investigation is that cultural differences in clinical and at-risk samples have been not explored despite potentially informative, *e.g.*, by exploring the differences between community and at-risk internationally adopted children who have different cultural backgrounds.

## Discussion

This opinion deepened two open questions raised by current literature on Family Drawing with attachment-based coding systems leading to ABCD classifications (Kaplan and Main, 1986) or scales (Fury, 1996).

The first question was about psychometric properties. Current findings highlight the main strength of FD in the inter-rater reliability, almost always reported and with good values for both systems across different samples. Instead, the scarcity of studies suggests a great need for research on discriminant validity. Particularly, it appears important to investigate if the results obtained with the FD are independent of the child’s ability to draw, enlarging the potential number of children assessable. If the investigation is routed concerning IQ, on which anyway more studies are needed, there is still a lot to do concerning the ability to draw. Few efforts focused on fine motor skills as a measurable parameter of the ability to draw, to understand if the FD runs the risk to classify as secure children more able to drawn, and less able children are more likely to be classified as insecure. However, a child’s drawing abilities depend on different skills besides fine motor ability, the topic which is still uninvestigated, *e.g.*, visuospatial skills (Toomela, 2002). One option can be to use a tool for evaluating drawing abilities (*e.g.*, Clark, 1995), including an evaluation of the same child’s abilities in drawing different contents, such as the family and another not-attachment-related topic, to also check if the content of the FD may elicit emotional arousal impacting visual-sensory skills (Costanzi et al., 2019).

Besides, drawing abilities varied according to gender and age (Wright and Black, 2013). Their influence should be further investigated, especially given that scarcity of available data hindered a meta-analytic investigation of their role in a study by Pace et al. (2021). This would help to understand whether gender differences suggested by some findings reflect those recognized in the wider literature on attachment, or whether the drawing ability is influential. Concerning age, existing studies included children in large age ranges, while more research on clustering age groups would help to define the optimal age range where the FD led to more reliable and accurate results.

Concerning concurrent validity, contrasting findings, and limitations of the few existing studies seem to suggest the informative utility to design mixed-method studies employing the SSP and/or the MCAST with the FD, analyzed with both coding systems and possibly including either community, clinical, or at-risk samples. In this regard, authors of studies employing a double method are also encouraged to publish data on convergent validity, *e.g.*, SSP and FD (Fihrer and McMahon, 2009).

The second research question inquired about the culture-fair potential of the FD. On the one side, results with both scoring systems support the universality of pattern B, as expressed without marked differences between Western and non-Western children (Mesman et al., 2016). On the other side, unexpected results raised doubts about the universality of indicators of insecure patterns, particularly the insecure-preoccupied pattern. These indicators of insecurity are based on a typically Western conception of parent–child dyads, and they could not capture the contribution of multiple sources of attachment security typical of some non-Western cultures where multiple adults contribute to raising children (e.g., African countries like Cameroon; Eloundou-Enyegue and Shapiro, 2004; Amos, 2013). In this case, the FD has the potential to leave the child free to draw all the significant figures he/she considers part of his/her family. However, it poses the problem of how to compare the results with those obtained with other measures based on the dyad, *e.g.*, SSP and MCAST.

Beyond inter-country differences, the FD would help in those situations where the reliance on a child’s verbal abilities is limited, *e.g.*, inter-country adopted or migrant or asylum seeker or refugee children, or clinical ones, *e.g*., children with selective mutism or social anxiety.

However, all these enthusiastic purposes urge to be substantiated by future investigations providing empirical support or disconfirming the FD culture-fair potential, for instance, through inter-country investigations or with mixed-method studies designed as proposed above, specifically selecting the previously mentioned subgroups of children as at-risk and clinical participants.

In conclusion, the convoluted and heterogeneous state-of-art of research on FD with attachment-related systems is probably due to a lack of continuous development and control of coding systems starting from the same developers, which led to multiple adaptations of the coding system (*e.g.*, Crittenden’s model in Carr-Hopkins et al., 2017) and fragmented contributions affecting the recognition of FD potential. Hopefully, this Opinion Article provides a valuable resource and an important starting point to guide future lines of research to advance the knowledge on this topic.

## Author contributions

CP conceptualized, structured the opinion, revised, and rewrote some parts. SM and FV wrote the first draft of the manuscript and made all further revisions until the final version. FV had performed Table 1 based on CP’s indications. All authors contributed to the article and approved the submitted version.
